# A Trying Time

**Published:** 1875-02

**Authors:** 


### A TRYING TIME.

Everybody knows by this time that the greatest cause of modern times is now on trial in the city of Brooklyn. It is not our purpose to allude to it, except in one of its phases. We will not venture to assert that Mr. Beecher is the victim of a vain woman and an artful man, who have found it not difficult to weave a net-work of suspicious circumstances around the leading pulpit orator of America and the world. Our purpose is merely to call attention to the trial from a sanitary point of view. Here are hundreds of human beings penned up week after week in a room from which the outer air is energetically excluded. If we allow a fair proportion of oxygen for each individual, so that one person shall not be compelled to use the exhausted air of himself or another, we cannot honestly certify that the court-room in question contains air enough for more than one-tenth of the persons who daily crowd it. In other words, the daily sessions of six hours should be reduced to a trifle more than half an hour, or the vitality of the occupants of the room must be impaired. We have sought to learn whether the windows of the room are all thrown open each night after the adjournment, in order to secure a complete ventilation and purification of the place through the night, but we are told that no care is taken in this direction. The room cools off as the steam with which it is heated, gets low, and the attendants think—as do many persons who are blessed with reasonable intelligence—that a cold room is a ventilated room, than which no error can be so greivous. If the steam-pipes were torn out, and a capacious furnace put in the basement, its heating-chamber supplied with a great flue leading out into the pure air, so that the very air which did the warming would supply some food for the lungs, the evil would be remedied in part. But for a room of the size, occupied by six hundred feeders—as all who breathe are really feeding the body with a pabulum without which it would quickly die—should have an air-flue leading into it, fully ten feet square, and points of exit nearly as large. By such means the atmosphere would sustain life, and the danger of disease and death in the court-room would be greatly lessened.

In the cause at issue in Brooklyn, only two persons are even charged with misconduct, and it is but fair to say that only a small part of the community believe these two to be guilty of the offence charged. It seems hardly just to subject these two to the slow but sure poisoning which they are undergoing by their daily attendance in court; is it not, then, criminal to compel the court, the attorneys, the witnesses, the reporters, the spectators, to brave the pestilence in this pursuit of duty or interest ?

We do manage to worry through life in the face of a great many dangers. It is surprising how much we can compel our bodies to endure. We can swallow one poison, absorb another through the mucus membrane of the mouth, and inhale a half dozen others, and yet so wonderfully alert is nature to employ all the agents whose office it is to neutralize these poisons, that we live on, month after month, and may not know for a long time that we have done ourselves infinite wrong, and have permanently weakened our hold on life.

The picture now visible in Brooklyn, is not a pleasant one from any point of view. From a hygenic standpoint, it is horrible. But it is not unlike the picture which may be seen in all States of the Union, and in every county seat.

It is merely the ancient spectacle—so old that it seems venerable to the thoughtless—of a "court of justice" in which law of the highest type is set at utter defiance. It is simply a picture of a place in which individuals—clean and unclean—are stowed in such numbers, with reference to the existing air-space, as to render it wholly impossible that the atmosphere presented for the use of the lungs can be capable of sustaining them without injury, more than half an hour after the house is occupied. This is all. It is the picture of human life destroyed by poison, nothing more. It is murder, to be sure, but it is murder for which nobody is responsible. The regular attendants at such places get sick, but nobody is frightened. The majority will fight it through ; some will die in a few months, some in a year or two, and some will last longer; but all, without one exception, will be the worse for the poison taken from the unchanged and unpurified air of that Brooklyn court-room. It may not be written of many, as it has been of young Henry Ford, who died yesterday, that "his illness is thought to have been caused by the bad air in the court-room, where the Beecher trial is in progress," but thousands will remember this famous action of the man Tilton against the great preacher, as the beginning of the end to them.

---

TRUSTWORTHINESS.—There is not a function nor a position in life in which trustworthiness is not the most valuable characteristic. Genius, which sends careless work as often as noble effort, which is not to be trusted for time or punctuality; energies which are heroic on one occasion, then sink below the measure of a child's strength on another; love that burns like the sun to-day, and is dead and cold as a mere heap of ashes to-morrow—who cares for such gifts as these ? Beautiful as they are when they come, they are intrinsically worthless, because so entirely unstable; and qualities which have not half the show and shine of these have twice their value, because always at hand when wanted, always to be trusted in and relied on when they have work to do and responsibilities to fulfill. Yes, it is a grand quality; we know none grander. It is the very crown, the gathering in of so many notable virtues, which, without it, are of no account, that we might part with many a good gift bestowed by nature upon man, rather than with this which gives vitality to all—this supreme excellence of trustworthiness. With it a boor has his worth; without it a demigod his dangers and his valuelessness. It means everything that is solid, everything that is worthy of confidence, and no one is so great that he is not ennobled and made of value by its possession.

---

ICE IN DIPTHERIA —The theory of the cure of diptheria by ice is simple—possibly too simple to attract attention. It is merely that the temperature of the parts infested by the bacteria is kept so low that development of the destructive parasite is impossible, and it soon dies or becomes innocuous. The one thing essential in the ice-treatment is, that it shall be persistent and unceasing, so that the inflamed parts shall not have time to fairly recover from the chill, or have the blood forced in upon them. Nature does the rest, and quinine and iron are never needed; in fact, the patient does better without them.

---

To wipe your teeth with a towel in order to clean them is a good deal worse than nothing. Does any reader wish to know why ?

